# REM Sleep Behavior Disorder and Cognitive Functions in Parkinson’s Patients: A Systematic Review

**DOI:** 10.3390/jcm12237397

**Published:** 2023-11-29

**Authors:** Giulia Marafioti, Francesco Corallo, Davide Cardile, Giuseppe Di Lorenzo, Angelo Quartarone, Viviana Lo Buono

**Affiliations:** IRCCS Centro Neurolesi Bonino Pulejo, 98124 Messina, Italy; giulia.marafioti@irccsme.it (G.M.); francesco.corallo@irccsme.it (F.C.); giuseppe.dilorenzo@irccsm.it (G.D.L.); angelo.quartarone@irccsme.it (A.Q.); viviana.lobuono@irccsme.it (V.L.B.)

**Keywords:** Parkinson’s disease, REM sleep, cognitive impairment, neurorehabilitation

## Abstract

Sleep disorders, such as REM sleep behavior disorder (RBD) and excessive daytime sleepiness, are among the most common non-motor symptoms in subjects with Parkinson’s disease (PD). Sleep disorders have a major negative impact on the quality of life of patients and their caregivers. In addition, REM sleep behavior disorder is an important risk factor for cognitive impairment in PD. This systematic review was conducted on studies investigating the influence of RBD on cognitive performance in PD subjects. We searched the PubMed and Scopus databases, screened the references of the studies included, and reviewed articles for additional citations. From the first 244 publications, we included only 11 studies that met the search criteria. The results showed that sleep disorders in PD were associated with impaired executive functions, visual-constructive abilities, reduced attention, and episodic verbal memory, and could predict the possible risk of developing dementia.

## 1. Introduction

Parkinson’s disease (PD) is a neurodegenerative disorder with a slow but progressive evolution, affecting around 1% of people over the age of 60 [1]. PD is associated with the loss of dopaminergic neurons, which results in decreased levels of dopamine in the putamen of the dorsolateral striatum, producing dysfunctions of direct and indirect pathways of movement control that involve cortico-basal-thalamo-cortical circuits [2]. In addition to classic motor signs and symptoms, such as tremor, rigidity, and bradykinesia, PD is characterized by a wide range of non-motor symptoms, including autonomic disturbances, olfactory deficits, cognitive impairment, mood dysfunctions, and sleep disorders [3,4]. Sleep disorders are the most frequent non-motor symptoms in PD, usually increasing in frequency over the course of the disease and disability progression and negatively impact patients’ everyday functioning and their own and their caregivers’ quality of life [5]. Sleep disorders represent a rather large and heterogeneous group of pathologies, including nighttime insomnia, increased sleepiness, sleep fragmentation, reduced sleep efficiency, and rapid eye movement (REM) sleep behavior disorder (RBD) [6,7]. RBD is a parasomnia that occurs during REM sleep, and it is characterized by the loss of physiological REM sleep atonia, dream enactment behavior, and complex motor behavior or vocalizations, occurring at any time between transitioning to sleep and waking [8]. Idiopathic RBD is a potential prodromal marker of neurodegenerative synucleinopathies [9,10]; it is widespread among subjects with PD (33 to 50 percent), multiple system atrophy (80 to 95 percent), and dementia with Lewy bodies (80 percent) [11,12]. Environmental and behavioral risk factors for RBD largely overlap with those for PD. In several studies, it was interesting to observe a significant gender difference in patients with Parkinson’s disease with possible RBD, as men were more affected by this peculiar sleep disorder than women [13].

According to some research, RBD may be a critical indicator of a subset of PD characterized by a non-tremor-dominant motor subtype or a kinetic-rigid motor phenotype and symmetric disease. Furthermore, severe non-motor symptoms such as increased autonomic dysfunction and visual hallucinations appear to precede RBD [14]. Previous cross-sectional studies revealed a strong association between RBD and cognitive impairments, in subjects with PD and RBD [15]. Concerning cognitive status, it is estimated that around 73% of PD subjects present with mild cognitive impairment (MCI) [16,17] with alterations in attention/executive function, visuoconstructional abilities, and episodic verbal memory. PD-MCI may represent an early stage of cognitive decline and a risk factor for developing dementia and, thus, an intermediate state between normal cognition and dementia, similar to amnestic MCI in the context of developing Alzheimer’s disease (AD) [18]. Recent advances in our understanding of PD-MCI, however, suggest that PD-MCI is rather heterogeneous with different clinical phenotypes, rates of progression, and, possibly, underlying mechanisms [19]. PD subjects diagnosed with RBD exhibit poorer cognitive performance than those without RBD in global cognitive function, long-term verbal recall, long-term verbal recognition, generativity, inhibition, shifting, language, and visuospatial/constructional ability. Notably, the risk of dementia in PD seems to be increased by RBD [20,21,22]. Longitudinal studies on non-demented subjects with PD revealed that sleep disorders may represent an independent risk factor for cognitive impairment and dementia [23]; however, these studies did not explore the evolution of cognitive functioning in PD patients with or without sleep disorders [24].

Despite increased research over the past two decades, the knowledge and treatment of non-motor symptoms in PD lag far behind our knowledge and treatment of its motor features. This review focused on studies that investigated the effect of RBD on cognitive function in patients with PD.

## 2. Materials and Methods

This study is based on a review of published/publicly reported literature, so it did not require permission by an ethics committee. A systematic review was conducted according to PRISMA guidelines to investigate the correlation between PD, sleep disorders, and cognitive performance. The studies were identified by searching the PubMed and Scopus databases from 2001 onwards. The keywords for this search were “Parkinson’s disease, rem sleep, and cognitive impairment”. (‘Parkinson disease’ [MeSH Terms] OR Parkinson’s [Text Word]); (‘Sleep, rem’ [MeSH Terms] OR rem sleep [Text Word]); (‘Cognitive dysfunction’ [MeSH Terms] OR cognitive impairment [Text Word]). After removing all duplicates from the initial list, the articles were carefully evaluated based on their title, abstract and text. Quality assessment of studies involved were performed according to JBI Critical Appraisal Checklist for cross-sectional studies.

### 2.1. Inclusion Criteria

A study was included if it described or investigated the relationship between RBD and cognitive alteration in PD with standardized neuropsychological measures. Only articles written in English were included in the review.

### 2.2. Exclusion Criteria

A study was excluded if there was a lack of assessment of the relationship between RBD and cognitive alterations in PD. Case report and systematic, integrative or narrative reviews were also excluded, although their reference lists were reviewed and included if appropriate. All articles without a control group or in language other than English were excluded.

## 3. Results

**Figure 1:**
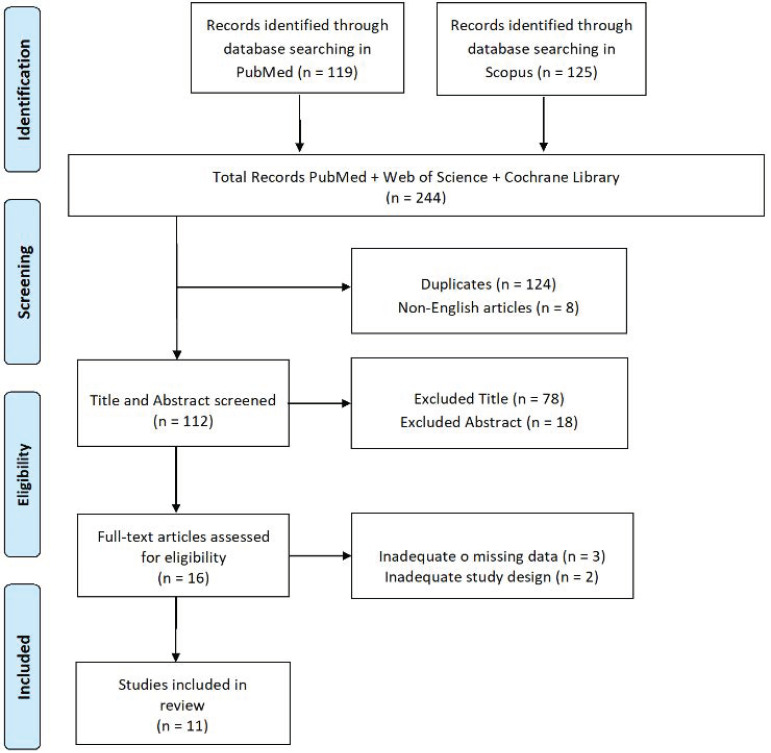
PRISMA flowchart.

Of the 244 articles identified, only 11 met the inclusion criteria (Figure 1). All studies investigated the influence of RDB on cognitive performance. Standardized tests used to evaluate sleep quality were: the Pittsburgh Sleep Quality Index (PSQI) [25], the Epworth Sleepiness Scale (ESS) [26], the REM Sleep Behavior Disorder Screening Questionnaire (RBDSQ) [27], the REM sleep behavior Hong Kong Disorder Questionnaire (RBD-HK) [28], the Test of Attentional Performance (TAP) [29], the Movement Disorder Society (MDS-UPDRS) [30], and the Parkinson’s Disease Sleep Scale (PDSS) [31].

The standardized tests used for cognitive assessment were as follows: the TAP, the Parkinson Neuropsychometric Dementia Assessment (PANDA) [32], the Mini–Mental State Examination (MMSE) [33], the Unified Parkinson’s Disease Rating Scale (UPDRS), the Movement Disorder Society (MDS) [34], the Montreal cognitive assessment (MoCA) [35], the Auditory verbal learning test (AVLT) [36], the Frontal Assessment Battery (FAB) [37], the Rey Auditory-Verbal Learning Test (RAVLT) [38], the Digit Span [39], the Trail Making Test A (TMT-A/B) [40], the Parkinson’s Disease Questionnaire (PDQ-39) [41], the Semantic Verbal Fluency Test (SVFT) [42], the Stroop Color Word Test (SCWT) [43].

All the studies conducted research on subjects with PD and evaluated the presence or absence of sleep disorders in 1.181 subjects of which: 864 PD, 139 with PD without dementia, 96 healthy subjects, 301 PD with RBD, 128 PD without RBD, 78 subjects with RBD, 62 patients who took MAO-B with other drugs, and 52 took antagonists and levodopa. The results for each study are summarized in Table 1.

To date, the influence of sleep disorders on cognitive function in patients with PD has been poorly investigated and poorly understood. What is well known is that sleep quality in PD is significantly correlated with cognition, and it differentially affects attention and executive function.

Hermann et al. [16] described significant differences in cognitive performance between patients with PD with and without RBD. Cognitive impairment, particularly deficits in attention, executive function, working memory, and semantic memory, has been associated with sleep quality disturbances and sleep breathing disorders. Furthermore, the authors have shown that excessive daytime sleepiness (EDS) is associated with depression, but there is no association with anxiety, apathy, or hallucinations. Finally, an association between EDS and the daily dose of levodopa (LEDD) was also found.

RBD and EDS are common disorders in patients with PD. In a study by Bjørnarå et al. [1], as opposed to excessive daytime sleepiness, RBD showed no association with disease duration or severity; however, PD patients with RBD reported more cognitive problems.

Gagnon et al. [20] found lower performance on visuoconstructional and visuoperceptual tests in PD patients with RBD compared with PD patients without RBD. This finding may be due to deficits in basic visual sensory functions, such as reduced acuity or contrast sensitivity, or in higher perceptual and constructional visual functions, which are commonly found even in early PD. The authors also suggest that RBD may be a risk factor for the development of dementia in PD.

The same results were reported by Zhang et al. [44], who described RBD as a significant independent risk factor for MCI. In their study, cognitive performance in verbal and visuospatial memory was worse in PD patients with RBD than in PD without RBD.

Kamble et al. [45] showed a more rapid cognitive decline in patients with PD with RBD than in those without RBD [46]. Patients with RBD were found to have significant impairment in many neuropsychological tests compared to RBD, such as category fluency, frontal assessment battery, attention, and verbal memory.

**Table 1:** Studies assessing sleep disorders and cognitive functions in Parkinson Disease.

Study	Sleep Quality Tools	Cognitive Deterioration Tools	Subjects	Outcomes
Bjørnarå KA, et al. [1]	RBDSQ PDSS MD-UPDRS	MD-UPDRS	107 patients with PD without dementia, of which 62 use MAO-B concomitantly with other drugs and 52 use a combination of antagonists and levodopa	The presence of cognitive decline as well as EDS is evident in patients with PD with and without pRBD.
Postuma RB, et al. [11]	RBD-HK	UPDRS MMSE MoCA MDS	61 of which 27 had basal RBD and 15 had no	The authors suggest that RBD in PD is associated with MCI.
Hermann W, et al. [16]	ESS PSQI TAP	PANDA	26 patients with PD with sleep disorders	The authors found significant differences in cognitive performance between patients with and without RBD, all associated with sleep quality disturbances and respiratory disturbances.
Gagnon JF, et al. [20]	ESS	DIGIT SPAN TMT- B RAVLT	32 patients with idiopathic RBD, 22 patients with PD with RBD, 18 patients with PD without RBD and 40 healthy control subjects	PD patients with RBD had more MCI than PD patients without RBD.
Zhang JR, et al. [44]	RBDSQ	DIGIT SPAN TMT-A TMT-B SCWT SVFT AVLT UPDRS	42 patients with PD without RBD, 32 with PD with RBD, 15 with iRBD, 36 healthy	Patients demonstrated clear associations between RBD symptoms and different PD-MCI domains.
Kamble N, et al. [45]	RBDSQ MSQ	MMSE MoCA FAB AVLT UPDRS	25 PD patients with RBD and 25 PD patients without RBD	In this study, the authors found many differences between PD patients with and without RBD, with different variations in sleep efficiency and cognitive decline being more rapid in PD patients with RBD.
Liu H, et al. [47]	RBDSQ PDSS-2	UPDRS MoCA	158 patients with PD of which 31 reported RBD	PD patients with RBD have high scores with respect to sleep and a large effect was also found on cognitive decline.
Bugalho P, et al. [48]	RBDSQ	MMSE UPDRS	75 patients with PD with RBD including 58 under dopaminergic treatment	The authors found high level of male PD patients with RBD, but found no significant differences in the scores obtained.
Nomura T, et al. [49]	RBDSQ	MMSE MoCA	70 patients with PD of which 19 with pRBD	PD patients with pRBD had higher levels of dementia than those without pRBD.
Yan YY, et al. [50]	RBD-HK	MMSE MoCA	89 patients with PD of which 46 with PD-RBD and 43 PD-npRBD	PD-pRBD patients demonstrate increased cognitive decline compared to PD-npRBD patients.
Suzuki K, et al. [51]	RBDSQ-J PDSS ESS PSQI	UPDRS PDQ-39	93 patients with PD and 93 control subjects with neurological or psychiatric diseases	In this study the authors found significant differences in PD patients with RBD showing high scores compared to the control group in the cognitive dimension which in turn affects sleep quality and vice versa.

Legend: PD: Parkinson’s disease; RBD: REM sleep behavior disorder; RBD: Idiopathic REM sleep behavior disorder; MCI: Mild Cognitive Impairment; PSQI: Pittsburg Sleep Quality Index; TAP: Test of Attentional Performance; ESS: Epworth Sleepiness Scale; RBDSQ: REM Sleep Behaviour Disorder Screening Questionnaire; UPDRS: Unified Parkinson’s Disease Rating Scale; MDS: Movement Disorders Society; FAB: Frontal cognitive dysfunction; PANDA: Parkinson Neuropsychometric Dementia Assessment; AVLT: Auditory Verbal Learning Test; MMSE: Mini-Mental State Examination; MOCA: Montreal cognitive assessment; SCWT: Stroop Color-Word Test; SDMT: Symbol Digit Modalities Test; SVFT: Semantic Verbal Fluency Test; TMT-A: Trail Making Test-A; TMT-B: Trail Making Test-B; RAVLT: Rey Auditory-Verbal Learning Test; MoCA: Montreal cognitive assessment; PDQ-39: Parkinson’s Disease Questionnaire; RBDSQ-J: RBD screening questionnaire; PDSS-2: PD Sleep Scale 2nd version; RBD-HK: RBD Questionnaire Hong-Kong; MSQ: Mayo sleep questionnaire.Influence of Sleep Alterations on Cognitive Functions.

Liu et al. [47] investigated the occurrence and clinical associations of RBD in patients with early-stage Parkinson’s disease. The authors confirmed the association between RBD and cognitive decline, particularly in the domains of attention, memory, and anxiety.

Contrary to some investigations, Bugalho et al. [48] analyzed PD patients in the early stage of disease and found no significant differences between patients with and without RBD on cognitive measures. As expected, in such early stages of the disease, they found only a very limited number of patients with global cognitive dysfunction.

In a 4-year prospective study, Postuma et al. [11] found that the presence of RBD predicted the eventual risk of developing dementia in patients with PD. RBD in PD was associated with impaired attention/executive functions, episodic verbal memory, and visuo-constructional abilities. In addition, in the study by Nomura et al. [49], PD patients with RBD presented more cognitive alterations than those without RBD, and the symptoms were associated with the development of dementia.

These data were confirmed by Yan et al. [50], who described severe cognitive impairment in PD with RBD compared with PD without RBD in the executive function, language, visuospatial ability, and attention domains, but, similarly, in the memory domain.

Suzuki et al. [51] showed that PD patients with RBD had impairment in visuospatial abilities and deficits in verbal memory, attention and executive function, and nighttime disturbances. Disease duration, motor scores and levodopa treatment did not differ between the PD with RBD and PD without RBD; however, RBD was associated with worse quality of life.

## 4. Discussion

Sleep is a complex phenomenon involved in various neurological and vegetative control functions and plays a crucial role in the development, processing, and maintenance of many aspects of psychological, cognitive, and motor functions. Sleep disorders have a significant impact on cognitive and physical functions and may be associated with psychological distress and depression. Sleep deprivation studies in normal subjects demonstrate that a lack of sleep can cause attention and working memory impairment. Moreover, untreated sleep disorders may present a significant risk factor for the development of cognitive impairment and significantly impair quality of life [52].

Sleep disturbances are commonly observed in a wide range of neurological conditions. Individuals with RBD may experience neurodegenerative illnesses, and these individuals are more likely to experience stroke in the future, indicating a possible link between RBD and stroke [53]. Sleep disorders represent frequent non-motor symptoms that seem to contribute significantly to everyday function impairment in subjects with PD [54]. Numerous forms of alterations of physiologic sleep patterns may be present at different stages during disease. The pathophysiology of sleep disturbances in PD involves degenerative changes and structural lesions in brainstem areas, in the dorsal midbrain and pons, that are related to sleep-wake activity and sleep regulation, or processes brought about secondarily, for example, by pharmacologic treatment [55].

Among sleep disorders, the literature highlights that RBD is one of the most important clinical markers and predictors of PD, and it may precede the onset of motor symptoms by several years [17,18,45,56,57]. In addition, the presence of RBD in PD patients is associated with a more severe subtype of the disease, with a strong negative prognostic with faster progression of both motor and non-motor symptoms [58].

Recently, Schenck et al. [59] reported that over 80% of men aged 50 years or older initially diagnosed with idiopathic RBD developed neurodegenerative disease, especially with alpha-synucleinopathy, including PD [60]. The high prevalence of RBD in PD may be explained by several common pathophysiological pathways, mainly related to the disruption of the mesolimbic dopamine circuitry [61].

Individuals with concomitant PD and RBD have more specific brain anatomical and functional changes compared to PD patients without RBD. Neuroimaging studies have reported brain dysfunctions associated with the presence of RBD in white matter integrity and reductions in gray matter volume in the pontomesencephalic tegmentum, medullary reticular formation, hypothalamus, thalamus, putamen, amygdala, and anterior cingulate cortex [32,62,63].

The data from the present review suggests that PD patients with RBD had greater cognitive impairment than those without RBD. Alterations in cognitive domains included deficits in executive function, visual-constructive abilities, reduced attention times, and episodic verbal memory [64]. Moreover, PD patients with RBD were more likely to have MCI and dementia [65,66].

The pathophysiological mechanism responsible for the association between RBD and cognitive alterations is still unclear, although there are several potential explanations. Longitudinal studies have shown that RBD may be an important risk factor for MCI in PD due neurotransmitter alterations, genetic mutation, neuroinflammation factors, altered cerebral metabolism and cortical activity slowing [67].

Several brainstem structures that are impaired in RBD or PD project to the cerebral cortex and modulate its neural activity. Alteration in this cerebral network seems to have an impact on cognitive functions. In addition, abnormal metabolic patterns with decreased perfusion in frontal and parietal areas, and increased perfusion in brainstem-cerebellar regions, were reported in PD with RBD and MCI [68,69].

Two distinct patterns of cognitive deficits have been identified in PD [70]. The first pattern describe posterior cortically based cognitive deficits associated with explicit memory and visuospatial alterations. The second pattern is related to fronto-striatal degeneration characterized by executive and attention deficit. It has been suggested that the dementia incidence in patients with PD is mainly associated with posterior cortical deficits. PD patients with RBD present a cognitive profile reflecting both frontostriatal and posterior cortical deficits [71].

Sleep disorders are conditions that affect the quality of life in PD subjects. A more profound insight into the underlying pathophysiological mechanisms intertwining sleep and neurodegeneration might lead to unique and individually tailored disease modifying or even neuroprotective therapeutic options in the long run. The correct management of sleep disorders in PD should start with sleep hygiene education and also include effective pharmacological treatments [72].

## 5. Conclusions

Literature data suggests an association between RBD and cognitive dysfunctions in PD patients. Patients diagnosed with RBD have specific cognitive and neuropsychiatric phenotypes characterized by poor global cognitive performance, but better long-term memory.

This systematic review focused on the lack of studies that explore the relationship between the impact of sleep disorders on cognitive functioning in PD. A small number of studies were included in this review because only 11 studies met the inclusion criteria. Furthermore, a meta-analysis was unable to be performed because quantitative information was not reported in the included studies.

Another limitation is the sample size: only a total number of 11,081 patients were included in the review. This contrasts with the high incidence of PD; therefore, the generalization of the results is limited. Despite these limitations, this descriptive review highlights important implications for the preventive management of sleep disturbance.

The exact mechanisms by which sleep behaviors are disrupted in PD are not entirely known; however, it appears that sleep alterations are directly related to the disease pathology. Clinical evidence seems to suggest that RBD is more than a simple sleep disorder. RBD is a well-known predictive clinical manifestation of neurodegenerative disease onset, including PD; therefore, an accurate evaluation of sleep disorders should be routinely evaluated in clinical settings.

## Data Availability

The data that support the findings of this study are available on request from the corresponding author.
